# Incidence and Clinical Relevance of COVID-19 in a Population of Young Competitive and Elite Football Players: A Retrospective Observational Study

**DOI:** 10.1186/s40798-022-00442-x

**Published:** 2022-04-15

**Authors:** Lidia Colangelo, Alessandra Volpe, Elisabetta Toso, Massimo Magnano, Mario Matta, Chiara Vignati, Andrea Marchini, Luca Semperboni, Luca Stefanini, Fiorenzo Gaita

**Affiliations:** 1 Department of Medical Sciences, University of Turin, Turin, Italy; 2 J Medical, Turin, Italy

## Abstract

**Background:**

The COVID-19 pandemic dramatically changed lifestyle worldwide, including sport. A comprehensive evaluation of the prevalence of cardiac involvement in COVID-19 is essential to finalize a safe protocol for resuming elite sport. The aim of this study is to evaluate incidence of cardiac involvement and COVID-19 impact on athletic performance.

**Materials and methods:**

This retrospective observational study analysed the data collected from consecutive competitive athletes who performed medical-sports examinations at the J Medical Center from March 2020 to March 2021. All athletes periodically performed a molecular test using a nasopharyngeal swab to detect COVID-19 infection. Positive athletes performed laboratory (cardiac troponin T—cTnT) and instrumental (echocardiography, stress test, Holter ECG) investigations following recovery to identify any cardiac involvement. Cardiac magnetic resonance imaging (MRI) was performed in case of abnormal findings at first-level evaluation.

**Results:**

Among 238 athletes (median age 20 years), 77 contracted COVID-19, mainly males (79%) with a median age of 16 years. Fifty-one athletes (66%) presented mild symptoms, and none required hospitalization. Evaluation for resuming sport was performed after a median of 30 days from the first positive test. Abnormal findings were obtained in 13 cases (5 athletes [6%] with elevated cTnT values; 13 athletes [17%] with arrhythmias on Holter ECG and/or during stress test; 2 athletes [3%] anomalies at echocardiography). Cardiac MRI discovered abnormalities in 9 cases, but none of these was clearly related to COVID-19 and none fulfilled acute myocarditis criteria. No negative impact on athletic performance was observed, and none of the athletes developed persistent COVID-related symptoms.

**Conclusions:**

Our registry confirms the predominantly self-limiting illness in young athlete population. The incidence of clear COVID-19-related structural myocardial injury was very low, but transient exertional ventricular arrhythmias or pericardial effusion was observed without significant impact on athletic performance. Implemented screening for return to activity is likely reasonable only in moderate-to-severe symptomatic athletes.

## Key Points

- This retrospective observational study confirms the predominantly self-limiting illness of COVID-19 in young, multi-ethnic athlete population, without high incidence of myocardial injury or significant impact on athletic performance.
- Transient exertional ventricular arrhythmias or pericardial effusion could be observed after COVID-19 infection; therefore, a usual return-to-play protocol evaluation must be performed after asymptomatic or mild symptomatic illness resolution.
- It is reasonable that higher risk of myocardial involvement could be observed in moderate-to-severe symptomatic athletes and further studies need to clarify if implemented screening for return to activity is necessary in this context.

## Introduction

Since it was discovered in China, coronavirus-related disease 2019 (COVID-19) has dramatically changed lifestyle worldwide introducing enormous changes in daily life, including sport activity. Many limitations such as cancelled competitive events, limited access to training facilities and sanitary restriction were adopted to contain the spread of the pandemic.

Coronaviruses belong to the large family of beta coronaviruses, only six of which (229E, NL63, OC43, HKUI, MERS-Cov, Sars-Cov-1) were previously known for their ability to infect humans [1]. The new coronavirus is responsible for severe respiratory disease, hence the name SARS-CoV-2 (severe acute respiratory syndrome coronavirus 2). The virus accesses host cells via the angiotensin-converting enzyme (ACE2), which is more abundant in alveolar type II cells of the lung. This receptor is also expressed in other tissues of the human body such as the heart, gastrointestinal tract, urinary tract, resulting in susceptibility to COVID-19 [2], with gravity related to density of ACE2 in each tissue. Due to these heterogenous virus’ targets, myocardial involvement represents one of the potential clinical or subclinical disease manifestation, being related to disease severity and worse prognosis in the general population [3–5].

Incidence, complications and performance consequences of COVID-19 in young athletes are not fully understood. The incidence of acute myocarditis in COVID-19 positive athletes is particularly relevant since the degree and type of sport activity can negatively affect the immunological process that often accompanies myocardial inflammation [6], resulting in the need of a de-training period of at least 6 months. This period of temporary "suspension" has a significant influence on young athletes’ performance. Before resumption of sport activity, it is essential that athletes who suffered COVID-19 infection are appropriately evaluated to rule out the presence of life-threatening cardiac conditions.

The purpose of our retrospective study was to evaluate the incidence of COVID-19 in young competitive and elite football players and the clinical implications that may have influenced performance and resumption of sport activity.

## Methods

### Study Population

We evaluated consecutive elite football players and young competitive non-professional athletes from Juventus Football Club who carried out medical-sports examinations at the J Medical Center of Turin from March 2020 to March 2021.

Elite professional athletes were defined as those practicing professional sport activity with continuity, in the context of the disciplines regulated by the Italian National Olympic Committee (CONI) (L.91/1981).

Competitive non-professional athletes were defined as those registered to national sports Federations recognized by CONI who, after sport-medical evaluation, obtain the certification for competitive fitness (DM 1982 and circular 7/1983) but whose activity is not considered professional.

All athletes, prior to the pandemic period, were examined according to the indications of the Ministry of Health and the Italian Sports Medical Federation (FMSI) to obtain eligibility (Table 1).

**Table 1:** Screening examinations proposed by the Ministry of Health and the Italian Sports Medical Federation (FMSI) and to obtain the eligibility of the competitive fitness

	FMSI protocol		Post-COVID protocol
	Professional athlete	Competitive non-professional athlete	
First-line examinations	Medical examination	Medical examination	Medical examination
	Resting ECG	Resting ECG	Resting ECG
	Treadmill stress testing	Treadmill stress testing	Treadmill stress testing
	Spirometry	Spirometry	Spirometry*
	Urinalysis	Urinalysis	Urinalysis
	Transthoracic echocardiography		Transthoracic echocardiography
	Blood test		Blood test (cTnT)*
			Holter Monitor ECG 24 h*
Second-line examinations	Cardiac MRI	Cardiac MRI	Cardiac MRI
	Holter Monitor ECG 24 h	Holter Monitor ECG 24 h	
		Blood test	
		Transthoracic echocardiography	

Cardiac MRI: cardiac magnetic resonance imaging; cTnT: cardiac Troponin T; ECG: electrocardiogram; FMSI: Italian Sports Medical Federation

*Not required but recommended

Because of the pandemic, the FMSI in agreement with the Ministry of Health on May 2020 issued a protocol to assess safe return to sport practice for all athletes following recovery from COVID-19 (Table 1).

COVID-19 infection was defined as a positive result of a real-time PCR (RT-PCR) rhino-pharyngeal swab test.

From July 2020, with the resumption of training, Juventus Football Club decided to carry out screening tests based on the Athlete’s category. Male athletes were categorized in:

- Elite professional athlete (EPA), aged > 16 years, whom professionalism is recognized by CONI and playing in the league at the top of the Italian football league system;
- Under 23 (U23), professional athletes with age predominantly < 23 years, playing in the third league of the Italian football league system;
- Under 19 (U19), non-professional athletes with age between 17 and 20 years;
- Under 17 (U17), non-professional athletes with age between 15 and 18 years;
- Under 16 (U16), non-professional athletes with age between 15 and 17 years;
- Under 15 (U15), non-professional athletes with age ≤ 15 years.

Female athletes were categorized in:

- EPA, aged > 16 years, whom professionalism is recognized by CONI and playing in the league at the top of the Italian football league system;
- Under 19 (U19), non-professional athletes with age between 16 and 20 years;
- Under 17 (U17), non-professional athletes with age between 15 and 18 years;
- Under 15 (U15), non-professional athletes with age ≤ 15 years.

From July 2020 to January 2021, 1 or 2 rhino-pharyngeal swabs (AllPlex-Seege CE-IVD, Seegene, Seoul, Republic of Korea) and serological tests searching for anti-SARS-CoV-2 antibodies (quantitative, chemiluminescence) of IgG and IgM class (m2000 SARS-CoV-2 assay, Abbott Laboratories, Illinois, USA) were performed every 14 days in professional athletes, while 1 rhino-pharyngeal test in case of close contact with a subject with positive results or suspicious symptoms was performed in competitive non-professional players. Since January 2021, all male and elite professional female athletes performed 1 rhino-pharyngeal test weekly. Non-professional female athletes underwent tests only in case of positive contacts or symptoms.

Athletes presenting positive RT-PCR test were suspended from sport activity until a negative test was obtained, following which clinical evaluation was carried out according to the recovery protocol provided by the Ministry of Health and FMSI, as presented below.

All patients gave written informed consent for participation in the study, which was approved by the institutional ethic committee and was performed according to the principles of the Declaration of Helsinki.

### Blood Chemistry

The following blood chemistry tests were considered: complete blood cells count with formula, C-reactive protein (CRP), ferritin, lactate dehydrogenase (LDH), interleukin 6 (IL-6), COVID IgG (cut-off for positive values > 7.1 BAU/mL), COVID IgM (cut-off for positive values > 1.9 BAU/mL) and cardiac troponin T (cTnT, normal value < 14 ng/L).

### Evaluation of Cardiac and Respiratory Function

Oxygen saturation was measured in all Athletes at rest and during exertion. Following the onset of desaturations or respiratory symptoms, spirometry and chest X-ray were performed as further investigations.

All Athletes underwent physical examination, resting ECG and stress testing according to the Master protocol.

Stress tests were performed on treadmill (PowerCube Ganshorn machine) using a Bruce protocol characterized by shorter (1–2 min) steps. During the test ECG abnormalities, arrhythmias and sport performance were analysed. Sport performance was evaluated with metabolic equivalent of task (METs), and the performance was assessed comparing these data with METs collected from a stress test performed before COVID-19 infection (ΔMETs).

In addition, cardiac morphology and function were evaluated using Color Doppler echocardiogram (GE Vivid E9 with XDclear), aiming to detect pathological changes in the cardiac structures related to COVID-19. The following evaluation were performed: left ventricular (LV) geometry assessed by end-diastolic volume (EDV); LV systolic function assessed by biplanar LV ejection fraction (EF) and regional wall motion; left atrium (LA) geometry assessed by end-systolic LA volume index (LAVI); right ventricular (RV) geometry assessed by RV basal diameter; RV systolic function assessed by the tricuspid annular plane excursion (TAPSE); estimated systolic pulmonary artery pressure (PAPs); search for pericardial effusion.

According to symptoms and/or abnormal results in previous examinations, Holter Monitoring (GE SEER 12 Digital Holter ECG Recorder machine) was performed in all Athletes, including a training session during the registration. Arrhythmias were categorized based on daily burden: not frequent, < 100 extra beats per day; frequent, between 100 and 500 extra beats per day; very frequent, > 500 extra beats per day. Repetitive ventricular arrhythmias, frequent or very frequent ventricular premature contractions, extra beats originating from left ventricle or uncommon repetitive supraventricular arrhythmias were considered worthy of further investigation.

In case of abnormal results, athletes could be referred for second level imaging investigation by means of cardiac magnetic resonance imaging (MRI) 1.5 T (Optima™ MR450w GEM). No general anaesthesia or sedation was required. Cardiac MRI included cine and T2-short tau inversion recovery (STIR) images (e.g. repetition time [TR] = 1154 ms, echo time [TE] = 102 ms), T2 mapping (e.g. TR = 612 ms, TE = 73 ms), and T1 mapping (e.g. TR = 3.3 ms, TE = 1.4 ms) before administration of contrast agents. Late gadolinium enhanced (LGE) 2D segmented inversion recovery sequences were acquired at 8 min after intravenous administration of contrast agent. Currently recommended criteria for diagnosis of acute myocarditis were assessed [7].

All these examinations were performed as soon as possible after the first negative rhino-pharyngeal test..

### Disease and Symptoms Severity Classification

According to the previous literature [8], the disease was classified as asymptomatic (positive SARS-CoV-2 test, no symptoms), mild (mild symptoms, no dyspnoea), moderate (clinical or radiographic evidence of lower respiratory tract disease, oxygen saturation ≥ 94%), severe (oxygen saturation < 94%, respiratory rate ≥ 30 breaths/min, lung infiltrates > 50%) or critical (respiratory failure, shock, and multiorgan dysfunction or failure). Asthenia, anosmia or ageusia, vomiting and/or diarrhoea, headache, cough and pharyngodynia, nasopharyngeal congestion, which persisted for a few hours or days with spontaneous regression, were all considered symptoms of mild degree.

### Statistical Analysis

Continuous variables, presented as means and standard deviations or median and interquartile ranges, were compared by Mann–Whitney’s test. Categorical variables, presented as absolute numbers and percentages, were compared using the chi-square test with Yates’ correction or Fisher’s exact test. For the comparison of data obtained from different groups, analysis of variance (ANOVA) was used, employing the Bonferroni correction. A two-sided *p* value < 0.05 was considered statistically significant. Statistical analyses were performed using SPSS 26.0 (SPSS, Chicago, Illinois, USA).

## Results

**Fig. 1:**
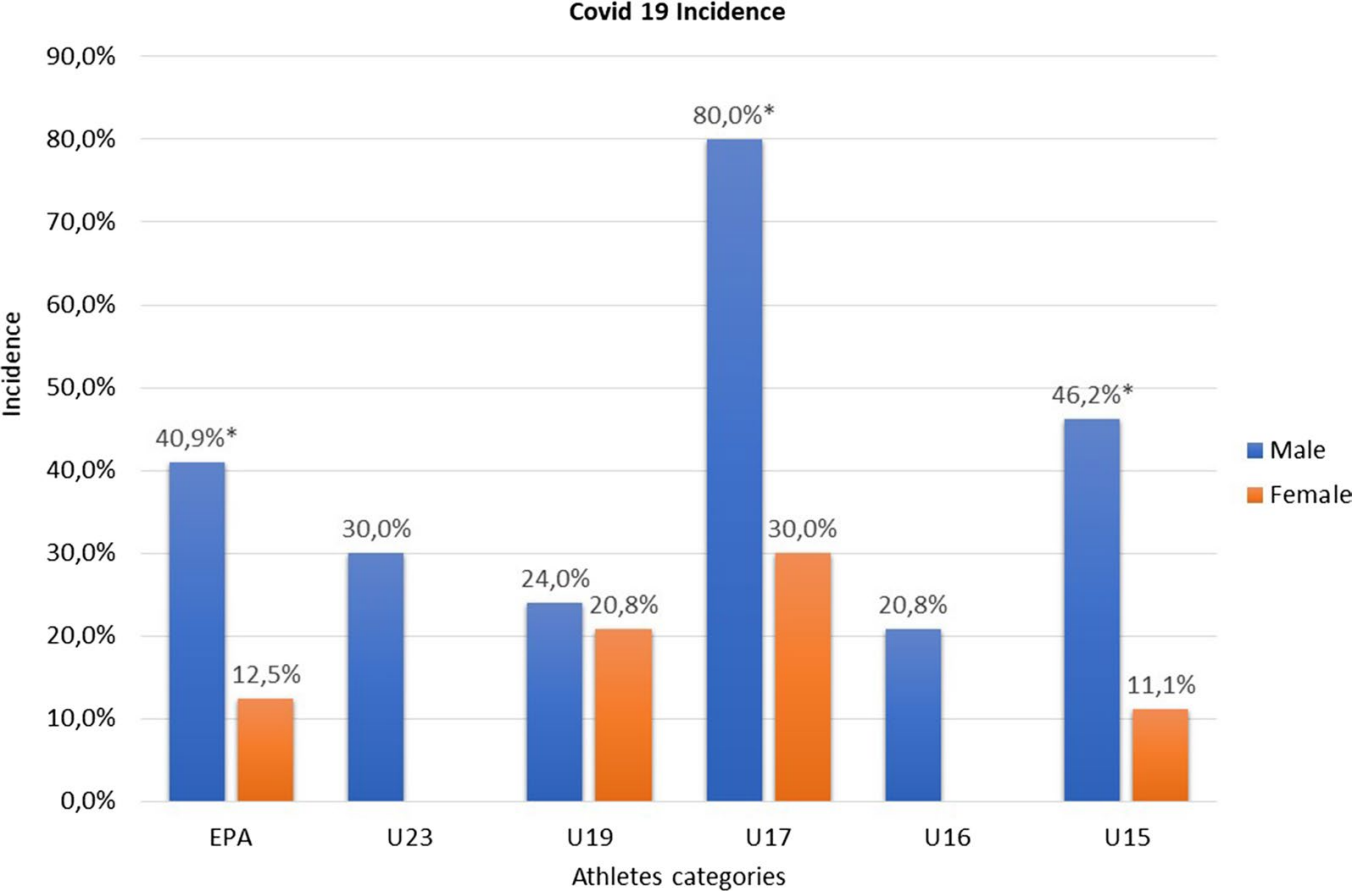
Differences in the incidence of COVID 19 by sex and football categories. EPA: elite professional athlete with age > 16 years; U23: professional athletes with age between predominantly < 23 years; U19: non-professional athletes with age 17–20 years; U17: non-professional athletes with age 15–18 years; U16: non-professional athletes with age 15–17 years; U15: non-professional athletes with age ≤ 15 years. *p value < 0.05

Among the total of 238 football players (101 elite professional athletes, median age 20 years, 152 males), 77 subjects (32%) contracted COVID-19 from March 2020 to March 2021. Among these, 61 (79%) were male and the median age was 16 years (range 15–18, average 18 ± 5). The incidence of the disease (Fig. 1 and Table 2) was significantly higher in male than in the female population (40% and 19%, respectively; *p* < 0.001) and it was predominantly distributed in the U17 category for both sexes (80% in male and 30% in female U17 players). The incidence was significantly higher in male than in female population even in analyses for categories in EPA group (41% and 12.5%, respectively, *p* = 0.04), U17 (80% and 30%, *p* < 0.01) and U15 (46% and 11%, *p* = 0.02).

**Table 2:** Clinical characteristics and medical assessments

Population	Total (238)	Male (152)	Female (86)	*p* value
**Clinical characteristics**				
Age (years)	19 ± 1	19 ± 1	18 ± 1	1
BMI (kg/m^2^)	22 ± 2	22 ± 1	21 ± 1	1
Positive tests	77 (32)	61 (40)	16 (19)	< 0.01*
**Clinical presentation COVID19**	**(77)**	**(61)**	**(16)**	
Age (years)	18 ± 5	18 ± 5	16 ± 4	0.14
Incidence for categories				
EPA	12 (25)	9 (41)	3 (12.5)	0.04*
U23	8 (30)	8 (30)		
U19	10 (21)	6 (24)	4 (21)	1
U17	28 (57)	21 (80)	7 (30)	< 0.01*
U16	5 (21)	5 (21)		
U15	14 (31)	12 (46)	2 (11)	0.02*
BMI (kg/m^2^)	22 ± 2	22 ± 2	21 ± 1	1
Asymptomatic	26 (34)	21 (27)	5 (6)	1
Mild symptoms	51 (66)	40 (52)	11 (14)	1
Astenia	22	16	5	
Ageusia/anosmia	21	18	3	
Fever	19	16	2	
Pharyngodynia/rhinitis	21	16	5	
Headache	21	21	0	
Muscle pain	15	13	2	
Diarrhoea	3	3	0	
Moderate/severe symptoms	0	0	0	
**Screening test COVID19**				
RNA polymerase	77 (100)	61 (100)	16 (100)	1
Serological tests	47 (20)	44 (29)	3 (3)	< 0.01*
Time from RPS+ to RPS− (days)	23 ± 10	23 ± 11	20 ± 8	0.31
EPA	24 ± 12	22 ± 12	29 ± 6	0.36
U23	20 ± 4	20 ± 4		
U19	19 ± 9	24 ± 9	13 ± 4	0.05
U17	25 ± 13	27 ± 14	16 ± 5	0.05
U16	22 ± 9	22 ± 9		
U15	18 ± 8	18 ± 9	18 ± 3	1
Time from RPS− to RtPP (days)	14 ± 4	12 ± 3	17 ± 6	< 0.01*
EPA	6 ± 21	7 ± 12	2 ± 1	0.50
U23	2 ± 2	2 ± 2		
U19	7 ± 7	3 ± 2	13 ± 6^c^	< 0.01*
U17	16 ± 9 ^a^	12 ± 6^b^	28 ± 9^c^	< 0.01*
U16	10 ± 6	10 ± 6		
U15	24 ± 27 ^a^	26 ± 29^b^	17 ± 3	0.68
**“Return to play” protocol**				
Blood tests	68 (88)	56 (91)	12 (75)	0.08
Resting ECG	77 (100)	61 (100)	16 (100)	1
Echocardiography transthoracic	77 (100)	61 (100)	16 (100)	1
Holter Monitor ECG 24 h	72 (94)	60 (98)	12 (75)	< 0.01*
Treadmill stress testing	77 (100)	61 (100)	16 (100)	1
Cardiac MRI	13 (17)	11 (18)	2 (12)	0.73
**Abnormal results**				
Cardiac troponin T (61)	5 (8)	5 (10)	0	
Resting ECG	0	0	0	
Echocardiography transthoracic	2 (3)	0	2 (3)	
Holter Monitor ECG 24 h	8 (11)	8 (13)	0	
Treadmill stress testing	8 (11)	8 (13)	0	

Analysis of variance (ANOVA) was used to compare data obtained from different teams

BMI: body mass index; EPA: elite professional athlete with age > 16 years; EAT: ectopic atrial tachycardia; ECG: electrocardiogram; F: female; F-VEB: frequent ventricular extra beats; I-VEB: isolated ventricular extra beats; LBBB: left bundle brunch block morphology; LGE: late gadolinium enhancement; M: male; NF-VEB: not frequent ventricular extra beats; No: no abnormal finding; PE: pericardial effusion; RBBB: right bundle brunch block morphology; RPS + : positive rhino-pharyngeal swab; RPS-: negative rhino-pharyngeal swab; RtPP: return to play protocol; U23: professional athletes with age between predominantly < 23 years; U19: non-professional athletes with age 17–20 years; U17: non-professional athletes with age 15–18 years; U16: non-professional athletes with age 15–17 years; U15: non-professional athletes with age ≤ 15 years

^*^p value < 0.05

The bold divides different sections of the table

^a^Non-homogeneous subgroups in total population according to the Bonferroni correction

^b^Non-homogeneous subgroups in male population according to the Bonferroni correction

^c^Non-homogeneous subgroups in female population according to the Bonferroni correction

**Fig. 2:**
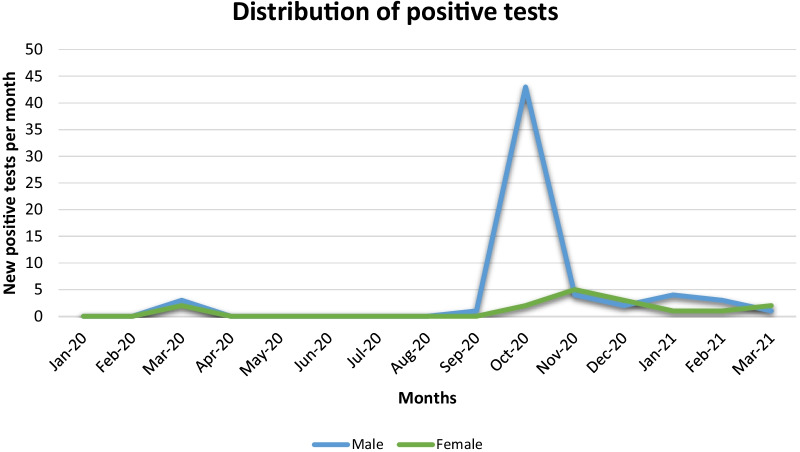
Time distribution of positive COVID-19 tests

The distribution of positive test over the study duration (Fig. 2) shows a first peak for both male and female athletes between February and April 2020 and a second peak significantly higher in male athletes between September and November 2020 (Fig. 2). This second peak was driven by a higher incidence of positive test in U 17 male athletes (89% of the positive test in this category was discovered in October 2020).

Fifty-one athletes (66%) presented symptoms, mainly of mild degree, while 26 (34%) were asymptomatic. None of the athletes developed severe symptoms such as desaturation or required hospitalization (Table 2). No differences between male and female players or between different team categories were observed in clinical presentation.

The median time between the first positive test and the negative one was 20 days (range 15–28, average 23 ± 10), and no differences between male and female (23 ± 10 and 20 ± 8, *p* = 0.3) or between team categories (*p* = 0.35) were observed. The return-to-play clinical evaluations were carried out on a median of 30 days after the first positive finding (range 24–41, mean 33 ± 17) and a median of 8 days after the negative result (range 2–15, mean 14 ± 4). Time from negative test to sport resumption was significantly higher in younger non-professional categories (16 ± 9 days in U17 players, CI 10–22, and 24 ± 27 in U15, CI 15–33, *p* = 0.01) and in female athletes than in male (17 ± 6 VS 12 ± 3, *p* < 0.001).

Due to changes in recovery protocol during the pandemic, only 68 of the 77 positive athletes underwent blood tests and the cTnT value was available in 61 of them. All athletes with symptomatic infection were subjected to blood tests, including cTnT concentration. Only 5 athletes (6%) had abnormal cTnT values with a mean value of abnormal cTnT of 28.9 ± 18.6 ng/L (median 20.1, range 19.3–36.3), while the remaining blood chemistry didn’t show any alteration, including inflammatory markers. Positive values of IgG were detected in 74% of young athletes after a median of 30 days after the positive RT-PCR; on the other hand, no positivity of IgM was highlighted (Table 3).

**Table 3:** Results of screening examinations for return to play post-COVID

Population	Male (61)	Female (16)
**Blood test**		
White blood cells (× 10^9^/L) (normal 4–10)	5.3 ± 1.1	7.2 ± 2.1
Lymphocytes (× 10^9^/L) (normal 1.1–4)	2.3 ± 5.1	2.3 ± 4.2
C-reactive protein (mg/dL) (normal < 0.5)	0.001 ± 0.001	0.001 ± 0.001
Ferritin (ng/mL) (normal 22–330)	78.7 ± 56	34.8 ± 20
LDH (mU/mL) (normal 80–300)	328 ± 61	264 ± 62
IL6 (pg/mL) (normal < 5.9)	1.07 ± 0.72	1.6 ± 1.3
COVID IgG (BAU/mL) (positive > 7.1)	34 ± 28	17.3 ± 8.8
COVID IgM (BAU/mL) (positive > 1.9)	0.46 ± 0.4	1.11 ± 0.8
cTnT (pg/mL) (normal < 14)	7.8 ± 10.4	3.4 ± 2
**Echocardiography**		
LV EDV (ml)	124 ± 23	95 ± 11
LV EF (%)	61 ± 9	65 ± 5
LAVI (ml/m^2^)	29 ± 5	26 ± 5
RV basal diameter (mm)	37 ± 4	34 ± 3
TAPSE (mm)	25 ± 4	24 ± 3
PAPs (mmHg)	24 ± 5	23 ± 3
Pericardial effusion	0	2
**Holter Monitor ECG 24 h**		
Heart rate (bpm)	67 ± 12	73 ± 10
Supraventricular ectopic beat	47	5
< 100/24 h	47	5
100–500/24 h	0	0
> 500/24 h	0	0
Ventricular ectopic beat	27	5
< 100/24 h	25	5
100–500/24 h	2	0
> 500/24 h	0	0
**Treadmill stress testing**		
METs pre-COVID	17.2 ± 2	15.1 ± 2
METs post-COVID	17.7 ± 1	15.5 ± 3
ΔMETs	+ 0.5 ± 0.2	+ 0.4 ± 0.3
Supraventricular ectopic beat	4	0
At rest	1	
Exertion	3	
Isolated	3	
Repetitive	1	
Ventricular ectopic beat	7	0
At rest	3	
Exertion	3	
Isolated	4	
Repetitive	2	
LBBB configuration	3	
RBBB configuration	4	

LV EDV: end-diastolic volume left ventricular, LV EF: left ventricular ejection fraction, LAVI: left atrium volume index, TAPSE: tricuspid annular plane systolic excursion, PAPs: pulmonary artery systolic pressure; MET: metabolic equivalent of task; LBBB: left bundle branch block; RBBB: right bundle branch block

No athlete showed any abnormalities on the resting ECG or desaturations at rest or during the stress testing.

Abnormalities during instrumental examinations were recorded in 13 athletes (17%). Isolated supraventricular arrhythmias were recorded in 3 male athletes during treadmill test. Ectopic atrial tachycardia triggered by exertion was recorded in one athlete in whom subsequently an electrophysiological study was performed to rule out re-entry supraventricular arrhythmias; no abnormalities at the endocardial mapping were highlighted. Holter monitoring highlighted not frequent isolated supraventricular extra beats in 52 athletes (47 male and 5 female), that were considered as non-pathological finding.

Isolated ventricular ectopic beats were recorded in 4 male athletes during stress testing. Repetitive ventricular ectopic beats triggered by exertion were registered in two male athletes: the first athlete presented a monomorphic ventricular couplet (RR’ 290 ms); the second one presented a monomorphic triplet (RR’ 260 ms). High ventricular ectopic beats burden (> 100/24 h) and/or repetitive ventricular ectopic beats (couplets and/or triplets) were highlighted in 8 male athletes by Holter monitoring, 4 of them with left ventricular origin (right bundle brunch block morphology). No repetitive ventricular arrhythmias were recorded by stress test before COVID-19 pandemic in any enrolled Athlete.

Considering sport performance by treadmill test, non-significant variation of METs has been registered after COVID infection. Among male athletes, the increase of METs post-infection was not significant (17.7 ± 1 vs 17.2 ± 2, *p* = 0.08), with a ΔMETs of + 0.5 ± 0.2. Among female players, METs post-COVID were non-significantly higher than METs pre-COVID (15.5 ± 3 vs 15.1 ± 2, *p* = 0.66), with a ΔMETs of + 0.4 ± 0.3. No difference in ΔMETs was observed between sexes (*p* = 0.12).

Mean left ventricular ejection fraction on echocardiography was 62% ± 4, with normal biventricular volumes (left ventricular EDV mean 59 mL/m2 in males, 53 mL/m2 in females; right ventricle basal diameter 37 mm in males, 34 mm in females). The echocardiography was normal in 73 athletes. In 2 female athletes (3%) were registered mild pericardial effusion and/or increased echogenicity of the pericardial sheets.

Cardiac MRI was performed in 13 athletes (17% of the overall positive tests, 3 of them asymptomatic) due to abnormalities observed during Holter monitoring, treadmill test and/or echocardiography. These MRI pointed out abnormalities in 9 athletes (12% of the COVID-19 positive): mild pericardial effusions in 4 athletes; late gadolinium enhancement (LGE) in 4 athlete, two of them presenting non-specific LGE in the junctional area, one in the intramyocardial area between the inferior interventricular septum and the inferior middle wall, one in the subepicardial area at the level of the infero-lateral wall; altered biventricular morphology in one athlete (biventricular hypertrabeculation), deeded not related to COVID-19. Criteria for the diagnosis of acute myocarditis were never reached; no myocardial oedema was found. No correlation was observed between cTnT values and LGE presence at cardiac MRI: patients with abnormal LGE areas showed normal values of cTnT at serological test.

Detailed description of clinical and instrumental abnormal findings in COVID-19 athletes is summarized in Table 4.

**Table 4:** Detailed description of clinical and instrumental abnormal findings in COVID-19 athletes

Patient	Category	Age	Sex	Symptoms	Stress ECG	Holter ECG 24 h	Echo	cTnT	Cardiac MRI
1)	EPA	28	M	No	I-VEB LBBB	No	No	5.6	PE + intramyocardial LGE
2)	EPA	27	M	Fever	No	No	No	62.8*	No
3)	EPA	35	M	No	No	No	No	8.1	NP
4)	EPA	37	M	No	No	No	No	19.8*	No
5)	EPA	23	M	Pharyngodynia	No	NF-VEB LBBB	No	6.6	Junctional LGE
6)	EPA	22	M	Asthenia	No	No	No	4.8	NP
7)	U23	22	M	Fever + pharyngodynia	R-VEB (triplet) RBBB	F-VEB RBBB	No	7.3	Subepicardial LGE
8)	U19	18	M	Fever + asthenia + headache + ageusia	No	NF-VEB	No	41.6*	PE + biventricular hypertrabeculation
9)	U19	19	M	Fever + asthenia + headache	I-VEB LBBB	No	No	4.7	NP
10)	U17	18	M	No	EAT	NF-VEB	No	4.6	PE
11)	U17	17	M	Fever + asthenia + headache	No	No	No	19.1*	No
12)	U17	18	M	Pharyngodynia + ageusia	R-VEB (couple) RBBB	NF-VEB	No	2.1	Junctional LGE
13)	U17	18	M	Pharyngodynia + asthenia + ageusia	I-VEB LBBB	No	No	20.5*	No
14)	U17	17	M	No	I-VEB RBBB	NF-VEB	No	6.9	No
15)	U16	17	M	Pharyngodynia	I-VEB RBBB	F-VEB	No	11.2	No
16)	U15	16	M	Headache + ageusia	No	NF-VEB	No	3.9	NP
17)	EPA	19	F	Asthenia + ageusia	No	No	PE	2.3	PE
18)	U17	17	F	No	No	No	PE	4.6	PE

EPA: elite professional athlete with age > 16 years; EAT: ectopic atrial tachycardia; F: female; F-VEB: frequent ventricular extra beats; I-VEB: isolated ventricular extra beats; LBBB: left bundle brunch block morphology; LGE: late gadolinium enhancement; M: male; NF-VEB: not frequent ventricular extra beats; No: no abnormal finding; PE: pericardial effusion; RBBB: right bundle brunch block morphology; U23: 20–23 years; U19: 18–19 years; U17: 17 years; U16: 16 years; U15: ≤ 15 years; “NP": not performed

*Abnormal value

In consideration of exercise-induced arrhythmias and/or LGE, 4 athletes were temporarily suspended. At 3-month follow-up, arrhythmias persisted despite detraining and specific therapy only in one Athlete who suffered from both ventricular repetitive arrhythmias from left ventricle and subepicardial LGE at cardiac MRI, while complete recovery and sport readmission occurred in the other 3 athletes.

## Discussion

The COVID-19 pandemic has substantially changed daily life all over the world, including sport. Several studies focused on the impact of this disease in athletes, who could be limited in their performance by prolonged respiratory symptoms or myocardial involvement. Return to sport activity after an adequate period of exercise restriction is another important aspect that has been focused by the previous literature [9–11].

According to the data observed in the general population [12], this study shows that the incidence of COVID-19 in young competitive athletes is relatively high (32%), with higher incidence in males compared to the female gender (male/female ratio about 2:1) [13], independently by the age categories; a relatively high incidence of asymptomatic infection (34%) discovered by regular monitoring in the absence of symptoms, has been reported in this study.

The higher incidence of COVID-19 in age < 17 years could be explained by the resumption of school lessons after the Italian lock-down [14]. This evidence is confirmed by the greater peak of the COVID-19 incidence registered in October 2020, despite the training and team meetings of the 2020/2021 season started between July 2020 and August 2020. Nevertheless, close contacts during training and matches between athletes certainly promote the transmission of the infection within a team. Therefore, close periodical screening is crucial in young athletes to detect at the earliest an epidemic outbreak.

In line with previous studies about the athletes’ population [15–18], our register confirms that COVID-19 in young competitive athletes presents predominantly with mild symptoms and self-limiting illness, without the need for hospital care. This finding is likely explained by the young age of the population and the absence of significant comorbidities [19, 20]. Differently from the results obtained by Krzywański et al. [17], in our registry no difference was observed in clinical presentation based on gender: the incidence of asymptomatic infection was the same in female than in male athletes, and this may be due to the younger age of our population compared to other studies (median age 16 years, range 15–18).

Patients who have experienced COVID-19 may develop chronic symptoms and/or a significant cardiorespiratory compromise that appear to have a pronounced impact on full athletic recovery [21–23]; this risk in athletes’ population had been estimated about the 18–27% [16, 17]. The potential risk of developing a persistent form of the disease was assessed in our study by the treadmill test. The METs reached during treadmill test performed following recovery from COVID-19 were non-inferior than the METs reached before the COVID infection, showing that athletes did not suffer persistent symptoms. Hull et al. [16] described lower respiratory tract symptoms and presence of chest pain as significant risk factors for “long COVID” development. In our population, none of the athletes presented pneumonia or chest pain. This finding could explain why in our study COVID-19 present limited impact on athletic performance recovery.

The decision concerning return to sport activity is challenging, and cardiac involvement should be ruled out after COVID-19 infection due to the potential risk of myocardial damage. Cardiac injury related to COVID-19 is mediated by the ability of the virus to directly, or indirectly, cause myocardial damage through multiple and synergistic mechanisms. In fact, by selective binding to ACE2 receptor, COVID-19 can cause disease in several tissues, especially where the enzyme is most expressed, as the heart itself [24]. Moreover, the virus induces the release of proinflammatory cytokines with a consequent toxic effect on various tissues, including myocardial cells, resulting in increased incidence of arrhythmias [25–27]. Concerning that point, a preliminary study conducted by Puntmann et all [28], which analysed the outcomes of COVID19 in an older population of non-athletes, showed myocarditis criteria in the 15% of the population. Despite this finding was at first confirmed in athletes’ population by Rajpal et al. [29], several subsequent studies supposed the incidental nature of cardiac injury detected at cardiac MRI in athletes, downsizing the real incidence of myocarditis from 15 to 0.6–3% [30–33]. Based on these findings, the current consensus recommends performing a second level cardiac imaging test only if an abnormal result is found by first-level examinations, or in athletes with persistent COVID-19 symptoms [10, 11]. According to this in our study cardiac MRI was performed only in 17% of the population, after abnormal results highlighted by Holter ECG monitoring and/or treadmill test (repetitive and/or frequent ventricular arrhythmias), echocardiography (pericardial effusion) and/or serological test (abnormal values of cTnT). Monomorphic ventricular extra beats, especially with left bundle brunch block morphology and inferior axis (originating from the outflow tract), are usually physiological findings in healthy athletes [34], being therefore non-discriminant in the decision to perform cardiac MRI. Interestingly, none of cardiac injuries that have been found in our population fulfilled the criteria for diagnosis of acute myocarditis. Furthermore, non-specific small LGE areas were found in 2 patients, representing a common finding in athlete population: it is well known that intense sport activity can lead to non-ischemic foci of fibrosis, not significantly related to potentially fatal ventricular arrhythmias [35, 36]. Similar data in elite athletes recovered from COVID-19 were reported by Malek et al. [37]: no signs of acute myocarditis were found, but 19% of athletes had some minor abnormalities at cardiac MR.

As previously reported [38, 39], pericardial late enhancements and/or pericardial effusion is one of the most common myocardial injury associated with COVID-19 infection. In our study, 4 asymptomatic mild pericardial effusion were observed, but none of these athletes showed significant symptoms or a decline in sport performance during exercise test.

As reported in previous studies [4, 5, 40], increased concentrations of cardiac troponin could be observed in general population affected by COVID-19, and it has been associated with disease severity and poor prognosis [5]. The same results have not been confirmed by other studies on athletes [29, 30]. According to the low disease severity and young age of our population, a modest increase in cTnT concentration was observed only in 6% of the athletes enrolled. Furthermore, the clinical significance of this observation is controversial due to the lack of specificity of increased cTnT levels in athletes: this marker can rise in response to exercise [41, 42]. Therefore, it is reasonable to not include cTnT blood test in routine cardiovascular screening after SARS-CoV-2 infection before returning to sport activity [10].

Interestingly, in our population only one player suffered significant myocardial abnormalities leading to suspension from competitive sport. The Athlete underwent cardiac MRI, showing left ventricle subepicardial LGE and persistent ventricular arrhythmias coming from the same area of left ventricle; troponin levels were normal, and no clear signs of acute myocarditis were detected at cardiac MRI. Therefore, it is not possible to establish sure correlation between these findings and COVID-19.

According to Cavigli et al. [43] who described uncommon ventricular arrhythmias and pericardial involvement with low incidence of myocarditis in young adult competitive athletes, the cardiac involvement in our study was predominantly characterized by arrhythmias and/or pericardial transient damage. This is probably related to the systemic inflammation following the infection, and not to a direct and persistent myocardial injury. Serological test in the present study could not confirm this sentence: no increase in white blood cells or CRP elevation was observed, differently from previous studies [44, 45]. However, blood samples were taken 23 ± 10 days (mean and standard deviation) after a positive rhino-pharyngeal swab, and any alterations might have already been resolved.

It is known that antibodies directed against SARS-CoV-2 can be detected in over 90% of subjects after 14 days since the onset of symptoms [46]. In our study, antibody response was observed in 74% of enrolled athletes irrespectively by age, gender or symptoms.

International guidance regarding protocols for sport resumption [10, 47] recommends at least 10 days between symptom onset and start of graduated exercise progression over the further 7 days. Nevertheless, given the rapidly emerging understanding of COVID-19, these recommendations are subject to adjustments as new evidence becomes available. Several studies highlighted the low incidence of cardiopulmonary complication of SARS-CoV-2 infection in young athletes; based on this low risk, shorter duration of sport interruption and less extensive screening before returning to competitive sport may appear reasonable in absence of severe disease or persistent exertional symptoms [16, 18]. A standard screening for competitive sport eligibility is likely sufficient, but particular attention must be paid to the search for any transient ventricular arrhythmias that may still be registered a few days after the resolution of the disease.

### Limitations

This study is limited by its retrospective, observational design, lacking comparison with a control population, since athletes with negative serological or molecular tests were not included. A second limitation is related to the time interval from the disease and the tests, which were performed only after the first negative test, which in most cases was obtained several weeks following the first positive test. This may therefore have influenced the low incidence of cardiological involvement highlighted in our case series. Additionally, the limited sample size of our population may not have allowed us to see any complication related to COVID19 due to the low incidence determined by the young age.

## Conclusions

This study reported a high incidence of COVID-19 infection in very young competitive football players. The higher incidence was found in men, especially school-age athletes. The prevalence of symptoms was high, but COVID-19 resulted in a mild, self-limiting illness in this cohort. Considering the clinical implications, cardiac involvement appears to be poorly represented and no acute myocarditis was observed. Prevalent cardiac presentations were non-clinically significant mild pericardial effusion and transient ventricular arrhythmias.

Our analysis confirms that implemented routine cardiorespiratory and haematological screening after COVID-19 infection in young athletes may be excessive. Standard cardiovascular evaluation for participation in competitive sport appears to be sufficient in asymptomatic or mildly symptomatic athletes, while more caution seems reasonable in moderately or severely symptomatic athletes following COVID-19 resolution.

## Data Availability

The datasets used and/or analysed during the current study are available from the corresponding author on reasonable request.
